# Colonic Angiosarcoma Arising in Association with Amyloid Deposits

**DOI:** 10.1155/2020/3780763

**Published:** 2020-05-16

**Authors:** Samar Said, Rondell P. Graham, Tsung-Teh Wu, Saba Yasir, Lizhi Zhang, Andrew L. Folpe

**Affiliations:** Department of Laboratory Medicine and Pathology, Mayo Clinic, Rochester, MN, USA

## Abstract

Angiosarcoma of the colon is rare, as is colonic amyloidosis. To our knowledge, there have been no reported cases of angiosarcoma arising in association with amyloid deposition. Herein, we described a case of 77-year-old man who presented with hematochezia, and a sigmoid mass was found on colonoscopy. Histologic examination of the resected specimen showed extensive nodular deposition of AL-lambda amyloid material in the colonic wall, as well as high-grade angiosarcoma which was closely intermingled with the amyloid deposits. While the occurrence of both colonic amyloidosis and angiosarcoma in this patient may represent pure coincidence, given the intimate association of the angiosarcoma and the amyloid deposition and the rarity of both of these lesions, we hypothesize that angiosarcoma could be secondary to amyloid deposition.

## 1. Introduction

Angiosarcoma is a rare malignant vascular neoplasm that involves mainly skin and superficial soft tissue and is extremely rare in the gastrointestinal tract. Most sporadic cutaneous angiosarcomas arise in the setting of significant sun damage and are thought to be related to long-term UV radiation damage [1]. Another important subset of cutaneous angiosarcomas arises following therapeutic radiation or lymphedema, most often in the setting of breast carcinoma [2]. A small number of angiosarcomas have been reported to arise in association with foreign materials, including shrapnel, Dacron vascular grafts, orthopedic prosthetic hardware, and tophaceous gout. Colonic amyloidosis is also rare, and most cases are immunoglobulin light chain-related. It is well known that some types of amyloidosis can be caused by malignancies. For example, calcitonin type amyloid deposits are caused by medullary thyroid carcinoma, and immunoglobulin type amyloidosis can be secondary to plasma cell dyscrasia or lymphoma. The association between sarcoma and amyloidosis was described in only few case reports. However, to our knowledge, there are no reported cases of angiosarcoma arising in association with amyloid deposition. Herein, we report an extremely rare example of colonic angiosarcoma arising in association with nodular amyloid deposits.

## 2. Case Presentation

**Figure 1:**
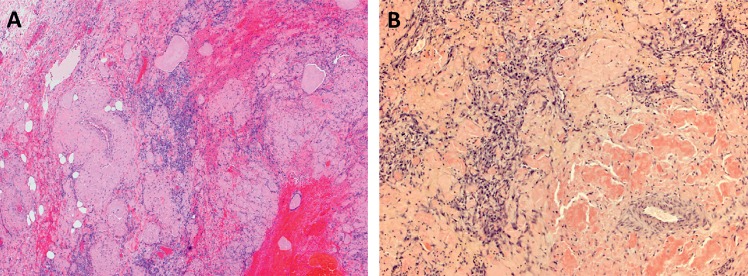
(a) Representative low-power image showing sheets of malignant cells and hemorrhage present between areas of extensive amyloid deposition (×40). (b) The amyloid deposits are Congo-Red positive (×100).

**Figure 2:**
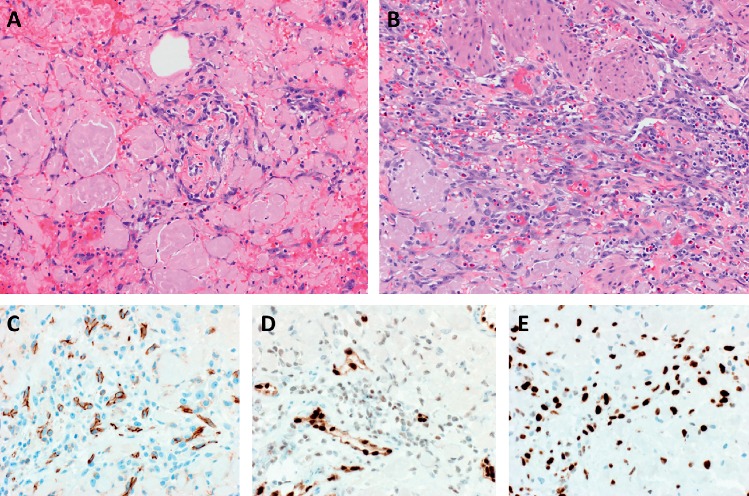
(a, b) Diffuse infiltration of amyloid deposits by high-grade angiosarcoma (×200). (c–e) The malignant cells are positive for CD31 (patchy) (c), ERG (d), and MYC (e) (×400).

**Figure 3:**
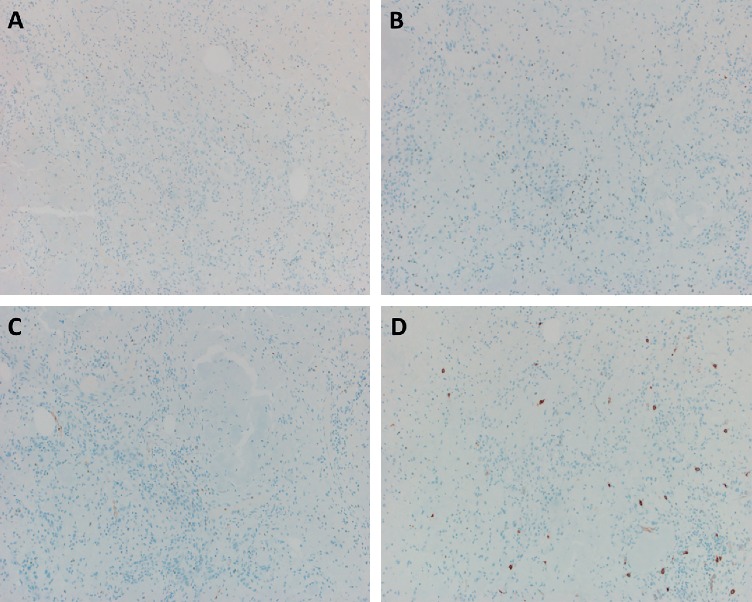
(a–d) The tumor cells are negative for keratin AE1/AE3 (a), SOX10 (b), S100 (c), and KIT (d) (×100).

A 77-year-old man with history of dementia, chronic kidney disease, gout, malignant melanoma of the skin, osteoarthritis, and gastroesophageal reflux disease presented with hematochezia. A nearly completely obstructing mass lesion in the sigmoid colon was found on colonoscopy. Follow-up staging CT scan of chest, abdomen, and pelvis showed a discrete mass in the distal sigmoid colon measuring approximately 10 cm in length, severe colonic wall thickening involving the distal colon and extending through the rectum, and a 2.5 cm right upper pole renal mass with heterogeneous enhancement worrisome for renal cell carcinoma. The patient underwent open sigmoidectomy. Grossly, there was a 3.4 × 2.0 × 0.5 cm ulcerated mass with raised borders. Sections submitted from the mass demonstrated extensive deposition of amorphous, pale eosinophilic material involving the lamina propria, muscularis mucosa, submucosa, muscularis propria, and subserosa with prominent involvement of vessels (Figure 1(a)). The material was positive with a Congo red stain (Figure 1(b)) and showed apple-green birefringence under polarized light, characteristic of amyloid. Closely intermingled with and diffusely infiltrating the amyloid deposits, there were sheets of highly atypical, plump spindled cells with amphophilic cytoplasm, oval large nuclei, prominent nucleoli, and frequent mitotic figures (Figures 2(a) and 2(b)). Intracytoplasmic vacuoles containing red blood cells were identified in some of the tumor cells. There were also areas of hemorrhage and blood lakes. The tumor cells were positive for endothelial markers including CD31 (patchy), ERG, and FLI1, confirming the diagnosis of angiosarcoma (Figures 2(c) and 2(d)) and negative for keratins (AE1/AE3), SOX10, S100 protein, and KIT (Figures 3(a)–3(d)). Strong expression of MYC protein by immunohistochemistry was present (Figure 2(e)). Fluorescence in situ hybridization (FISH) showed no evidence of MYC gene amplification.

Amyloid typing by laser microdissection (LMD) followed by high-performance liquid chromatography and tandem mass spectrometry (LC-MS/MS) confirmed the presence of amyloid and was consistent with AL (lambda)-type. No infiltration of plasma cells in the vicinity of amyloid deposits was seen. Due to the patient's advanced dementia, the family decided not to pursue aggressive therapy and opted for palliative care.

## 3. Discussion

Angiosarcoma is a rare malignant vascular neoplasm that involves mainly skin and superficial soft tissue but may occur in large vessels, deep soft tissue, and parenchymal organs such as the liver, spleen, bone, breast, and heart. It can be primary or secondary. Long-term sun exposure, radiation, chronic lymphedema, and exposure to Thorotrast, polyvinylchloride, and arsenic are known risk factors [1–5]. Angiosarcoma has also reported in association with arteriovenous fistulas in patients with renal failure [6–12], arthroplasty [13, 14], and injection sites [15] and in association with foreign materials that can be introduced to the body during trauma, surgery, and injection or formed in situ. Foreign materials reported to cause angiosarcoma include metals (such as bullets, shrapnel, cardiac defibrillator implants, and orthopedic joint prostheses) [16–20], Dacron graft [18, 21], silicone breast implants [22–24], retained surgical material such as sponge [25–27], and a gouty tophus (where it was hypothesized that the angiosarcoma was induced by the gouty tophus in a mechanism similar to that of foreign body-associated sarcoma) [28]. While development of sarcoma in association with foreign material occurs only rarely in humans, it is a well-documented phenomenon in rodents in which investigators have demonstrated the development of sarcoma after insertion of foreign material [29]. Different types of sarcoma have been reported in this setting including undifferentiated sarcoma, angiosarcoma, osteosarcoma, and chondrosarcoma. There is usually a prolonged latency period between the initial exposure to the foreign material and development of sarcoma [30]. The physical properties of the material seem to play a role in tumorogenesis, as the tumorigenic effect is reduced or lost when the foreign materials are inserted in pulverized or shredded forms, when their surface is rough or when they contain large pores. Formation of thick fibrous capsule around the foreign material is thought to play a role in tumorgenesis as tumorigenic material developed more fibrosis than the nontumorigenic material [30, 31]. MYC amplification, and strong MYC protein expression, initially thought to be limited to secondary angiosarcoma (such as postradiation and lymphedema-associated angiosarcoma) [32], is now known to occur in primary angiosarcoma as well [33].

Colonic angiosarcoma is extremely rare. Wang et al. reported a case of colonic angiosarcoma and summarized the results of 33 previously reported cases [34]. The mean age was 56 years (range 16 to 85 years), and the mean tumor size was 5 cm (range 1.5–12 cm). In more than two thirds of the cases, the tumor was in the left colon (36% in the sigmoid colon and 33% in the anorectal region), whereas 21% were in the cecum, 9% in the ascending colon, and 3% in the transverse and descending colon. Four of the 33 patients had a history of radiation exposure, and in one patient the tumor was associated with retained surgical gauze. An association with renal transplantation, long-term hemodialysis, and chronic colorectal ulcers were also reported (one case each). Clinical symptoms included gastrointestinal bleeding (67%), abdominal/anal pain (46%), intestinal obstruction (24%), diarrhea (50%), and weight loss (18%). Anemia was present in 42% of patients. Prognosis was poor with a median survival of 4.5 months. Tumor size less than 5 cm and younger age were associated with better prognosis. In general, colonic angiosarcomas are treated surgically, although there may also be a role for adjuvant therapies, including radiation, chemotherapy, and immunotherapy. Gastrointestinal amyloidosis is also rare. Cowan et al. [35] reported 76 patients with gastrointestinal amyloidosis (accounted for 3.2% of patients with amyloidosis), most of which (80%) were due to immunoglobulin light chain. The median age was 61 years (range 34 to 79 years). The stomach was the most affected site involved in 49% of cases followed by the colorectal region (46%), small bowel (42%), and esophagus (8%). Clinical symptoms included weight loss (43%), gastrointestinal bleeding (37%), heartburn (33%), early satiety (33%), diarrhea (29%), and abdominal pain (28%). The amyloidosis was systemic in 79% of cases and localized (i.e., without evidence of an underlying plasma cell dyscrasia or other organ involvement) in 21%. Supportive care was the main treatment for localized gastrointestinal amyloidosis with all patients alive at a median follow-up of 36 months. Patients with systemic AL amyloidosis were treated with anti-plasma cell chemotherapy with 72% survival rate at 4 years.

Amyloid has multiple precursor proteins and can occur in different settings including chronic inflammatory conditions, certain neoplasms, genetic/inherited, and age-related. Hematologic malignancies and medullary thyroid carcinoma are two neoplasms that are known to cause amyloid deposition where medullary thyroid carcinoma causes calcitonin type amyloid and plasma cell dyscrasia and some types of lymphoma can cause immunoglobulin related amyloidosis [36]. Amyloid deposition has also been reported in association with other epithelial neoplasms including urothelial carcinoma, neuroendocrine tumors, pituitary adenoma, gastric carcinoma, nasopharyngeal carcinoma, breast carcinoma, and endometrioid carcinoma and rarely in association with sarcomas including Kaposi's sarcoma, follicular dendritic cell sarcoma, leiomyosarcoma, and pleomorphic sarcoma [37–56]. The relationship between the amyloid and most of the previously mentioned neoplasms, however, is largely unknown. To our knowledge, this is the first case of angiosarcoma arising in association with amyloid deposition. This may represent a purely coincidental occurrence. However, given the intimate association of the angiosarcoma and the amyloid deposition and the rarity of both colonic angiosarcoma and colonic amyloidosis, angiosarcoma could be secondary to the amyloid deposition. It is unlikely that the amyloidosis was secondary to the angiosarcoma, as the area of amyloid deposition was considerably larger than the area involved by angiosarcoma, angiosarcoma was present only within areas of the colon also involved by amyloid, and amyloid deposition has never been previously reported in association with this tumor type. Furthermore, angiosarcomas are rapidly growing, aggressive tumors that usually come quickly to clinical attention. Perhaps most importantly, the amyloidosis type was AL (lambda)-type, which is not known to arise from solid malignancies but rather results from production of amyloidogenic light chains from a clonal plasma cell population. Other factors such as chronic illness or genetic predisposition might have contributed to this exceptional occurrence. AL amyloidosis is the most common type of both systemic and localized amyloidosis. In localized AL amyloidosis, the amyloidogenic light chain is thought to arise from local plasma cell populations, but histologic evidence of monoclonal plasma cell infiltration or lymphoma adjacent to amyloid deposits is not always present [57] (as in this case). Unfortunately, the patient's family decided against further workup, and thus we do not know if his amyloidosis was systemic or localized.

In summary, we report an extremely rare occurrence of a high-grade colonic angiosarcoma arising in close association with colonic amyloidosis. Although the exact relationship between the tumor and amyloid is obscure, it is tempting to speculate that the amyloid deposits may have served as the inciting tumorigenic event, although pure coincident cannot be completely excluded.
